# Serum Levels of Vitamin A and Vitamin D and Their Association With Symptoms in Children With Attention Deficit Hyperactivity Disorder

**DOI:** 10.3389/fpsyt.2020.599958

**Published:** 2020-11-23

**Authors:** Hong-Hua Li, Xiao-Jing Yue, Cheng-Xin Wang, Jun-Yan Feng, Bing Wang, Fei-Yong Jia

**Affiliations:** ^1^Department of Developmental and Behavioral Pediatrics, The First Hospital of Jilin University, Changchun, China; ^2^Pediatric Research Institute of Jilin Province, Changchun, China

**Keywords:** attention deficit hyperactivity disorder, vitamin A, vitamin D, dopamine, children

## Abstract

**Objective:** To measure levels of vitamin A (VA) and vitamin D (VD) and the symptomatic association of their co-deficiencies on attention deficit hyperactivity disorder (ADHD) in Chinese children (6–9 years).

**Methods:** Eighty-two children (69 boys and 13 girls; mean age = 7.1 ± 0.9 years at the time of the diagnosis) with ADHD were recruited as ADHD group. A total of 106 healthy children were recruited as the healthy control (HC) group. Serum levels of retinol and 25-hydroxyvitamin D (25(OH)D) of all children were evaluated using high-performance liquid chromatography (HPLC) and HPLC-tandem mass spectrometry. The Swanson, Nolan, and Pelham IV Rating Scale (SNAP-IV) was employed to assess the clinical symptoms of ADHD.

**Results:** Children suffering from ADHD had significantly reduced serum levels of retinol and 25(OH)D compared with those of HCs, and the prevalence of VA deficiency and VD deficiency were higher in children suffering from ADHD. Serum concentrations of 25(OH)D and retinol were linked closely with the presence or absence of ADHD after adjustment for age, body mass index, season of blood sampling, and sun exposure. Serum concentrations of 25(OH)D and retinol showed a negative correlation with the total scores of SNAP-IV. Children with ADHD as well as VA and VD co-deficiency had increased SNAP-IV total scores and ADHD inattention subscale scores.

**Conclusion:** VA deficiency and VD deficiency in children with ADHD were increased in comparison with that in HCs. VA and VD co-deficiency associated with ADHD symptom severity. Attention should be paid to regular testing of VA levels and VD levels. However, the mechanism of VA and VD in ADHD needs to be further studied. Interventional studies on VA and VD supplementation are recommended to further verify the relationship between VA and VD co-deficiency and ADHD.

## Introduction

Attention deficit hyperactivity disorder (ADHD) is considered to be the most common childhood-onset neurodevelopmental ailment (1). In ADHD, hyperactivity, impulsivity, and impaired attention are increased, and are inappropriate to the level of development in children (1). Among children, the global estimate of ADHD prevalence is 7.2% (2). Approximately 65% of children suffering from ADHD will have symptoms and impairment throughout adolescence and into adulthood (3). These symptoms can cause varying degrees of damage to the academic achievement, daily life, social interactions, and well-being of children suffering from ADHD.

The exact etiology of ADHD is not known, but has been postulated to occur as a result of the interplay of multiple psychological and biological factors. The impact of the environment and nutrition might lead to epigenetic changes (e.g., histone modification, DNA methylation) and may make a key contribution to ADHD development (4–6). Micronutrients can promote the differentiation and development of cells, and mediate the proliferation, apoptosis, and neurotransmission of neurons. Deficiency of some micronutrients, such as vitamin D (VD), during pregnancy and postnatal development might have some adverse effects upon brain activity and other vital functions (4). Studies have shown that low maternal levels of VD during pregnancy can lead to an increased risk of offspring with ADHD (7, 8). Studies have shown that children suffering from ADHD have a reduced serum concentration of 25-hydroxyvitamin D (25(OH)D) as compared with that in healthy children (9–11). VD supplementation is an adjunct therapy used to overcome symptoms of ADHD and has comparatively few side effects (12).

Genetic and environmental studies have implicated dysfunction of the dopamine system in ADHD pathogenesis (13). Methylphenidate is a psychomotor stimulant used commonly against ADHD. Methylphenidate has a therapeutic role because it can improve dopamine signaling in the prefrontal cortex and hippocampus (14). VD is a neuroactive steroid that provides protection to the dopamine system via regulation of gene expression (15). Moreover, VD binds to the VD receptor and modulates the expression of catechol-O-methyltransferase and tyrosine hydroxylase on a transcriptional level, the key enzymes assisting the production and metabolism of dopamine (16, 17). Hence, VD might have a role in the ADHD pathogenesis.

Vitamin A (VA) deficiency is widespread in developing countries, especially in pregnant women and children (18). Studies by the United Nations Children's Fund on global VA deficiency have shown that ~140 million children are living with VA deficiency (19). VA is an essential micronutrient. Retinoic acid (an active form of VA) is associated with the growth, development, and differentiation of cells in the central nervous system. *In vivo* studies have shown that VA deficiency damages the plasticity of hippocampal synapses and impairs spatial learning and working memory, and that these actions are mediated by retinoic acid nuclear receptor-α (20, 21). The cognitive impairment observed in ADHD patients may be related to a deficit in synaptic-plasticity mechanisms. Abnormal synaptic plasticity in the hippocampus can be found in a prenatal nicotine-exposure model of ADHD in mice (22). Furthermore, VA and VD have synergistic roles in regulating gene expression (23). In the nucleus, VD and retinoic acid must bind to the VD receptor (VDR) and retinoic acid receptor (RXR) complex to have a role in gene regulation (24). Therefore, VA deficiency might make a key contribution to ADHD pathogenesis.

Some authors have reported a correlation between VD deficiency and ADHD (9–11) but others have shown no significant variations between VD levels in ADHD children and those of healthy children (25). Only one research report has focused on the VD levels of ADHD children in China (26) and the relationship between VA levels and ADHD has not been reported.

Here, we measured levels of VA and VD in Chinese children (6–9 years) with ADHD and explored their symptomatic association with ADHD.

## Materials and Methods

### Ethical Approval of the Study Protocol

The Research Ethics Committee of the First Hospital of Jilin University (Changchun, China) approved the study protocol (2017-314). This study is registered in the Chinese Clinical Trial Registry (ChiCTR-OPC-17013502).

Participation in this case–control study was voluntary. Before recruitment, the parents of children suffering from ADHD provided written informed consent.

### Exclusion Criteria

The exclusion criteria were children: (i) with other neurodevelopmental or neurological disorders (intellectual disorders, autism, epilepsy, neurodegenerative disorders); (ii) with chronic diarrhea, malnutrition, or a history of micronutrient supplementation within the last 3 months; (iii) taking medications that may affect the metabolic process of VA and VD such as glucocorticoids, benzodiazepines and other drugs that may affect liver and kidney function.

### Enrolment

A group of children exhibiting signs of ADHD and who were being evaluated for the first time at the Child Developmental and Behavioral Division of the First Hospital of Jilin University from October 2018 to January 2020 were enrolled. Initially, all children with suspected ADHD were examined through reviews of their current health, developmental history, and family history, as well as through a physical examination and parental interviews carried out by a developmental pediatrician with reference to the *Diagnostic and Statistical Manual of Mental Disorders* (*DSM-5*) ADHD criteria (27). In addition, the Vanderbilt ADHD Diagnostic Rating Scale was used to evaluate comorbidities (e.g., learning disabilities, oppositional defiance disorder, conduct disorders).

### Study Cohort

Ultimately, 82 children (69 boys and 13 girls) fulfilled the ADHD criteria. The mean age of participants was 7.1 ± 0.9 years. Thirty-six participants presented with a predominantly inattentive type, five with a predominantly hyperactive type, and 41 with a combined type. Fourteen participants also had learning disabilities.

The healthy control (HC) group (*n* = 106; 79 boys and 27 girls; mean age: 7.1 ± 0.7 years) were recruited from Child Developmental and Behavioral Division of the First Hospital of Jilin University in the same period. They did not have the symptoms of neurodevelopmental disorders or other disorders associated with the disturbance of micronutrient homeostasis.

### Initial Clinical Assessment

The parents or caregivers of children in the ADHD and HC group completed a questionnaire created by our research team (on the basis of standardized guidelines) on demographic features (sex, age, place of residence, physical activity, sunlight exposure, daily diets, vitamin supplementation). An electronic scale and stadiometer were employed for measurement of the weight and height of participants in lightweight clothes with bare feet. We categorized children according to the age-percent distributions of the body mass index (BMI). Children with a BMI less than the 85th percentile were classified as “normal,” those with a BMI at the 85–95th percentile were considered “overweight,” and children with a BMI above the 95th percentile were categorized as “obese” (28).

### Behavioral Assessments

The Swanson, Nolan, and Pelham Rating Scale (SNAP-IV) is an unstructured questionnaire on behavior completed by a child's parent and teacher (29). The checklist features 26 items, covering three subsets of atypical behavior: inattention, hyperactivity/impulsivity, and oppositional defiant behaviors. Each item is graded on the basis of the symptom criteria, with a rating of 0 denoting “not at all,” 1 denoting “just a little,” 2 denoting “quite a bit,” and 3 denoting “very much.” The overall score for ADHD is obtained by adding the scores of inattention and hyperactivity/impulsivity subsets, with a higher score indicating an increased level of ADHD symptoms. The subscale scores are obtained by adding the scores of the items in the same subset. The Chinese version of SNAP-IV was used in the present study with significant psychometric characteristics (30).

### Laboratory Measurements

After an overnight fast, venous puncture was undertaken and a sample of peripheral blood (3 mL) was taken from each child, followed by serum isolation via centrifugation (within 6 h). Serum samples were stored at −20°C. Serum concentrations of retinol and 25(OH)D were measured via high-performance liquid chromatography (HPLC) and high-performance liquid chromatography–tandem mass spectrometry (HPLC–MS/MS) in the clinical laboratory of Jilin Hehe Medical Center (Changchun, China). The criteria used for evaluation of HPLC and HPLC–MS/MS have been discussed extensively in our previous work (31). A serum level of retinol (in μmol/L) <0.7, 0.7–1.05, and >1.05 was regarded as “VA-deficient,” “marginal VA,” and “normal VA,” respectively (32). A serum concentration (in ng/mL) of 25(OH)D of 30–90, 21–29, and <20 was regarded as “optimal VD,” “VD insufficiency,” and “VD deficiency,” respectively (33).

We divided the year into two periods: summer season (June–October), and winter season (November–May). This strategy has been found to be rational according to the results obtained in Boston (MA, USA) (34). Changchun and Boston are both between 42° and 45° of the northern latitude.

### Statistical Analyses

SPSS v22.0 (IBM, Armonk, NY, USA) was employed for analyses. The normality of the data distribution was evaluated using the Kolmogorov–Smirnov test. Continuous data are the mean ± SD or median percentile (P50), P25 or P75. Categorical data are given as frequencies with percentages. According to the distribution of the analyzed variable, an independent-sample *t*-test or a non-parametric Mann–Whitney *U*-test was employed to compare the continuous data of the two groups. A chi-square test was conducted to test the variation in the proportion of categorical variables among the two groups and *z* test was performed for pairwise comparisons. Spearman's correlation coefficient was employed for evaluation of the significance of correlation factors with the serum concentrations of retinol and 25(OH)D. The correlation between the serum concentrations of retinol and 25(OH)D and the presence or absence of ADHD was determined via multivariate logistic regression. The Kruskal–Wallis *H* test was used to compare the differences of the total and subscale score of SNAP-IV among three groups. The Bonferroni-adjusted significance test was used for pairwise comparisons. *P* < 0.05 (two-sided) was considered significant.

## Results

### Comparison of Sociodemographic Features Between the HC Group and ADHD Group

Table 1 shows the sociodemographic data of the two groups. Significant differences in sex, age, BMI, parents' level of education, residential area, season of blood collection, daily intake of dairy products, and sun exposure between children living with ADHD and HCs were not found.

**Table 1:** Comparison of sociodemographic features between the HC group and ADHD group^a^.

**Variable**	**ADHD**	**Control**	**χ^2^/*t/Z***	***P***
Number of participants	82	106		
Sex			2.189	0.139
Male	69 (84.1%)	79 (74.5%)		
Female	13 (15.9%)	27 (25.5%)		
Age (years)	7.1 ± 0.9	7.1 ± 0.7	0.037	0.971
BMI			1.946	0.378
<85th percentile	51 (62.2%)	70 (66.0%)		
85–95th percentile	15 (18.3%)	23 (21.7%)		
>95th percentile	16 (19.5%)	13 (12.3%)		
Father's level of education			0.002	0.962
High school or below	50 (60.9%)	65 (61.3%)		
University degree or above	32 (39.1%)	41 (38.7%)		
Mother's level of education			0.364	0.546
High school or below	50 (61.0%)	60 (56.6%)		
University degree or above	32 (39.0%)	46 (43.4%)		
Residence			0.098	0.754
Urban area	50 (61.0%)	67 (63.2%)		
Rural area	32 (39.0%)	39(36.8%)		
Season of blood collection			1.346	0.246
Winter	41 (50.0%)	62 (58.5%)		
Summer	41 (50.0%)	44 (41.5%)		
Annual household income (¥)			0.363	0.834
<100,000	20 (24.4%)	30 (28.3%)		
100,000–250,000	45 (54.9%)	55 (51.9%)		
>250,000	17 (20.7%)	21 (19.8%)		
Daily intake of dairy products (mL)	240 (200, 300)	245 (200, 300)	0.251	0.802
Daily exposure to sunlight (h)	3 (2, 3)	3 (2, 3)	0.114	0.909

a *n(%), mean ± SD, P50 (P25, P75)*.

*ADHD, attention deficit hyperactivity disorder; BMI, body mass index*.

### Serum Levels of VA and VD in the HC Group and ADHD Group

The serum concentrations of 25(OH)D and retinol in the two groups are outlined in Table 2. Compared with that in the HC group, the mean serum retinol level in children with ADHD was reduced significantly. In comparison with the HC group, the percentage of children with marginal VA deficiency in the ADHD group was increased significantly. The serum level of 25(OH)D in the ADHD group was reduced significantly compared with that in the HC group. In comparison with the HC group, the percentage of children with 25(OH)D deficiency in the ADHD group was increased significantly, whereas the percentage of children with an optimal 25(OH)D level in the ADHD group was reduced.

**Table 2:** Serum levels of retinol and 25(OH)D in the HC group and ADHD group^a^.

**Status**	**ADHD**	**Control**	***t/χ^2^***	***P***
Serum retinol (μmol/L)	1.09 ± 0.22^*^	1.21 ± 0.25	3.306	0.001
Normal	50 (61.0%)	80 (75.5%)	4.554	0.033
Marginal	32 (39.0%)^*^	26 (24.5%)		
25(OH)D (ng/mL)	19.4 ± 6.5^*^	24.0 ± 7.6	4.428	<0.001
Optimal	6 (7.3%)^*^	18 (17.0%)	7.363	0.025
Insufficient	33 (40.2%)	51 (48.1%)		
Deficient	43 (52.4%)^*^	37 (34.9%)		

a *n(%), mean ± SD*.

*ADHD, attention deficit hyperactivity disorder; 25(OH)D, 25-hydroxyvitamin D*.

* *P < 0.05 compared with the HC group*.

### Correlation Between Serum Levels of VA and VD With SNAP-IV Scores

The correlation coefficient for the serum levels of retinol and 25(OH)D and the SNAP-IV total score and SNAP-IV subscale score are shown in Table 3. The serum level of retinol was negatively correlated with the SNAP-IV total score. The 25(OH)D level had a negative association with the SNAP-IV total score and the subscale score of ADHD inattention.

**Table 3:** Correlation between serum concentrations of retinol and 25(OH)D with ADHD symptoms.

**SNAP-IV score**	**Serum level of retinol (μmol/L)**	**Serum level of 25(OH)D (ng/mL)**
Total of SNAP-IV	−0.23^*^	−0.25^*^
ADHD-IA	−0.19	−0.23^*^
ADHD-HI	−0.15	−0.21

*ADHD-IA, subscale score for inattention in SNAP-IV; ADHD-HI, subscale score for hyperactivity/impulsivity in SNAP-IV*.

* *P < 0.05*.

### Associations of the Serum Levels of VA and VD on the Presence or Absence of ADHD

Multivariate logistic regression analyses revealed the serum retinol level (adjusted odds ratio = 0.194; 95% confidence interval, 0.047–0.796; *P* = 0.023) and serum 25(OH)D level (0.912; 0.868–0.958; <0.001) to be associated significantly with or not associated with ADHD after adjustment for age, BMI, season of blood sampling and sun exposure (Table 4).

**Table 4:** The correlation between serum levels of retinol and 25(OH)D and presence or absence of ADHD.

**Variable**	**Adjusted OR (95%CI)**	***P***
Serum retinol (μmol/L)	0.194 (0.047–0.796)	0.023
Serum 25(OH)D (ng/mL)	0.912 (0.868–0.958)	<0.001
Age (years)	1.010 (0.977–1.044)	0.568
BMI		0.615
<85th percentile		
85–95th percentile	0.663 (0.259–1.695)	0.391
>95th percentile	0.584 (0.193–1.766)	0.341
Season of blood collection	0.643 (0.337–1.225)	0.179
Daily exposure to sunlight (h)	1.132 (0.723–1.772)	0.587

*OR, odds ratio; outcome variable, ADHD presence (1 = yes, 0 = no) (P < 0.05)*.

### Associations Between VA and VD Co-deficiency and SNAP-IV Scores

In the ADHD group, 76 children (93%) were found to have VA deficiency or VD deficiency. Of these children, 31 cases had VA and VD co-deficiency, 45 cases had VD deficiency only, 6 cases had normal VA and VD, and no cases had VA deficiency alone. There were significant differences in the total score of SNAP-IV and the subscale score of ADHD inattention between the VA and VD co-deficiency, the VD deficiency only and the normal VA and VD group. The SNAP-IV total score in the VA and VD co-deficiency group were increased significantly in comparison with those in the VA and VD normal group (Bonferroni-adjusted *P* = 0.047). The subscale score of ADHD inattention in the VA and VD co-deficiency group were increased significantly in comparison with those in the VD deficiency-only group (Bonferroni-adjusted *P* = 0.035) and the VA and VD normal group (Bonferroni-adjusted *P* = 0.014). (Table 5). No significant differences were found in the SNAP-IV total score and the subscale score of ADHD inattention between the VD deficiency-only group and the VA and VD normal group.

**Table 5:** Associations between VA and VD co-deficiency and SNAP-IV scores^a^.

**Scores**	**VA and VD co-deficiency (*n* = 31)**	**VD deficiency only (*n* = 45)**	**VA and VD normal (*n* = 6)**	***H***	***P***
Total of SNAP-IV	35 (31, 40)^*^	32 (27, 39)	30 (27, 31)	8.513	0.014
ADHD-IA	20 (18, 24)^#^	18 (16, 23)	16 (16, 17)	10.957	0.004
ADHD-HI	15 (12, 18)	14 (10, 17)	14 (11, 14)	2.470	0.291

a *P50 (P25, P75)*.

* *Bonferroni-adjusted P < 0.05 compared with the VA and VD normal group*.

# *Bonferroni-adjusted P < 0.05 compared with the VD deficiency only group and the VA and VD normal group*.

*ADHD-IA, subscale score for inattention in SNAP-IV; ADHD-HI, subscale score for hyperactivity/impulsivity in SNAP-IV*.

## Discussion

The current study elicited three main findings. First, children living with ADHD had significantly reduced serum levels of retinol and 25(OH)D than those of HCs, and that the prevalence of VA deficiency and VD deficiency was higher in ADHD children. Second, a negative correlation was found between serum levels of retinol and 25 (OH) D with the adverse symptoms of ADHD. Third, children with ADHD in the VA and VD co-deficiency group had increased SNAP-IV total scores and ADHD inattention subscale scores, which revealed that co-deficiency of VD and VA had a considerable association with ADHD symptoms in children.

VD is a neuroactive steroid associated with neuronal differentiation and development of the nervous system. Various studies have indicated that VD plays an important part in promoting normal development of the brain. Also, its role in neurotransmission alterations, neuro-immunomodulation, and antioxidation leading to mood disorders, neuropsychiatric and behavioral disease have been reviewed in various studies (35–37). According to data yielded from animal models, VD deficiency during development leads to brain-associated abnormalities and changes in multiple neurotransmitter (noradrenaline, serotonin, and dopamine) systems (35, 38).

Studies on VD and ADHD have been reported in recent years. Kamal et al. (39) conducted a case–control study involving 1,331 children and adolescents with ADHD aged 5–18 years. They found a high prevalence of VD deficiency in ADHD (>90%), which was significantly higher than that of HCs. Recently, Khoshbakht et al. (9) undertook a meta-analysis on VD levels and ADHD. They discovered that reduced VD levels were correlated significantly with the likelihood of ADHD, and that perinatal suboptimal VD concentrations were correlated significantly with a higher risk of ADHD in later life. In the present study, VD levels in children suffering from ADHD were reduced significantly in comparison with those of HCs, data which are consistent with the research mentioned above. However, the VD levels and prevalence of VD deficiency in ADHD children in our study are somewhat different from those in the studies mentioned above, which could be related to differences in sample size, age, geographic latitude, and dietary habits.

There is a lack of clarity regarding the key pathologic variations associated with ADHD. The dopaminergic dysfunction of the prefrontal cortex is associated with ADHD pathogenesis (13, 40, 41). VD promotes normal development and maturation of dopaminergic neurons by controlling expression of dopaminergic-associated genes (15, 17). VD affects the synthesis of growth factors such as glial cell-derived neurotrophic factor (GDNF) and neural growth factor, which are crucial for the survival of dopaminergic neurons (42), and also modulates GDNF/Ret signaling in dopaminergic neurons (43). In addition, VD has been found to be associated with regulation of expression of tyrosine hydroxylase (mediated by N-cadherin), which is the key enzyme for the synthesis and metabolism of dopamine (16). Our study and the data mentioned above reveal that VD deficiency might be associated with the pathology and occurrence of ADHD. Children with ADHD should be tested routinely for VD levels and given necessary supplementation.

Interestingly, we found that serum retinol levels in ADHD children were reduced significantly, and that the prevalence of marginal VA deficiency was ~40%, which was significantly higher than that of HCs. We reported, for the first time, the correlation of VA levels in children with ADHD. Retinoic acid is the active metabolite of VA, and makes a vital contribution in maintaining the normal function of the central nervous system. A deficit in synaptic plasticity may be related to the cognitive impairments observed in ADHD patients. In a prenatal model of ADHD in mice based on nicotine exposure, abnormalities in hippocampal synaptic plasticity and changes in glutamatergic signaling of hippocampal pyramidal neurons within the CA1 region have been found, which lead to impairment of learning, memory and attention (22, 44). Imaging studies on brain structures have revealed that children and adolescents suffering from ADHD have decreased intracranial and hippocampal volume (45). Retinoic acid binds to the RXR, and is implicated in hippocampal synaptic plasticity (46–48). *In vivo* studies have revealed that VA deficiency damages hippocampal synaptic plasticity and impairs spatial learning and working memory mediated by retinoic acid nuclear receptor-α (20, 21). Hence, we speculated that VA deficiency may have a key contribution to the pathophysiological mechanism of ADHD. Furthermore, VA and VD have a synergistic role in the nucleus; retinoic acid and VD must bind to the RXR and VDR complex to play a role in gene regulation (23, 24). Our study also showed that children with VA and VD co-deficiency had greater SNAP-IV total scores and ADHD inattention subscale scores, suggesting that co-deficiency of VA and VD aggravates ADHD symptoms. Our data also suggest that VD and VA co-deficiency may jointly participate in the pathophysiological process of ADHD. Therefore, regular testing of VD levels and VA levels could be recommended for children with ADHD.

Our study was case-controlled in design and, to avoid the influence of confounding factors on the levels of VA and VD, we matched the age, sex, season of blood collection, daily intake of dairy products and daily exposure to sunlight. Nevertheless, our study had three main limitations. First, eating behavior has a great impact on the nutritional status of children and thereby also on vitamin levels. There might be a possibility that children with ADHD may have different eating behaviors than HCs. “Picky” eaters and “partial” eaters may affect the serum levels of VA and VD: we did not analyze these effects. Second, in the questionnaire we created, the daily duration of sunshine exposure and daily intake of dairy products were reported mainly by parents. Therefore, exact matching in terms of these two variables for the two groups was not possible. The third limitation was the cross-sectional nature of the study without a time perspective. Thus, only associations between the vitamin concentrations and ADHD symptoms can be found, but not causality.

## Conclusions

VA deficiency and VD deficiency in children with ADHD were increased in comparison with that in HCs. VA and VD co-deficiency associated with ADHD symptom severity. Attention should be paid to regular testing of VA levels and VD levels. However, the mechanism of VA and VD in ADHD needs to be further studied. Interventional studies on VA and VD supplementation are recommended to further verify the relationship between VA and VD co-deficiency and ADHD.

## Data Availability Statement

The raw data supporting the conclusions of this article will be made available by the authors, without undue reservation.

## Ethics Statement

The studies involving human participants were reviewed and approved by The Research Ethics Committee of the First Hospital of Jilin University (Changchun, China). Written informed consent to participate in this study was provided by the participants' legal guardian/next of kin.

## Author Contributions

H-HL and X-JY designed the work, collected and analyzed the data, and drafted the manuscript. C-XW, J-YF, and BW collected and analyzed the data and revised the manuscript. F-YJ contributed to the concept and design of this study, reviewed, and revised the manuscript. All authors contributed to the article and approved the submitted version.

## Conflict of Interest

The authors declare that the research was conducted in the absence of any commercial or financial relationships that could be construed as a potential conflict of interest.
